# Assessment of the Diagnostic Potential of *miR-29a-3p* and *miR-92a-3p* as Circulatory Biomarkers in Acute Myeloid Leukemia

**DOI:** 10.31557/APJCP.2019.20.12.3625

**Published:** 2019

**Authors:** Marwa M Gado, Nahla O Mousa, M A Badawy, Maha A El Taweel, Ahmed Osman

**Affiliations:** 1 *Biotechnology/Biomolecular Chemistry Program, Department of Chemistry, Faculty of Science, *; 3 *Department of Chemistry, Faculty of Science, *; 4 *Clinical Pathology Department, National Cancer Institute, Cairo University, Giza, *; 2 *Biotechnology Program, Department of Biology, The American University in Cairo, *; 5 *Biochemistry Department, Faculty of Science, Ain Shams University, Abbasyia, Cairo,*; 6 *Biotechnology Program, Basic and Applied Sciences Institute, Egypt-Japan University of Science and Technology, Borg Al Arab, Alexandria, Egypt. *

**Keywords:** AML, miRNAs, miR-29a-3p, miR-92a-3p, qRT, PCR, MCL1

## Abstract

**Background:**

Acute myeloid leukemia (AML) is a set of Myeloproliferative neoplasms that are identified by excessive growth of myeloid blasts and production of abnormal blood cells. AML is the most common type of acute leukemia that occurs in adults. In addition, AML progresses rapidly and is considered a fatal disease. Thus, there is an urgent need to find new targets for molecularly designed therapies. In This study, we evaluated the circulatory levels of *microRNA-29a-3p (miR-29a-3p)* and *miR-92a-3p* beside exploring the expression pattern of their target gene myeloid cell leukemia sequence1 (*MCL1*) to investigate the role of these molecules in AML pathophysiology and to assess their ability to diagnose AML patients.

**Methods:**

40 adult AML patients along with 20 healthy subjects were enrolled in this study. Plasma were separated from venous blood samples, collected on EDTA, of all individuals were used to assess circulating miRNAs’ levels. In the meantime, total RNA was extracted from isolated leukocytes and was used to quantify target mRNA transcript levels.

**Results:**

Our data revealed that the circulating levels of *miR-29a-3p* and *miR-92a-3p *exhibited significant reduction in 90% and 100% of AML patients, respectively, when compared to the control group (p<0.001). On the other hand, the transcript level of the target gene of these miRNAs, *MCL1*, showed a sharp increase in 77.5% (p<0.001) of AML patients, along with a negative correlation with its regulatory *miRNAs*, *miR-29a-3p* and *miR-92a-3p*.

**Conclusion:**

Our data validates the negative regulatory role of *miR-29a-3p* and* miR-92a-3p* to the expression levels of MCL1 in peripheral blood and indicates that these miRNAs can be used as non-invasive diagnostic markers. Furthermore, our study highlights the therapeutic potential of *miR-29a-3p* and *miR-92a-3p* to target and downregulate a very important gene (*MCL1*), which is highly implicated in the pathogenesis of AML.

## Introduction

Acute myeloid leukemia (AML) comprises a set of myeloproliferative neoplasms, which are characterized by excessive growth of myeloid blasts and production of abnormal, immature blood cells (Jabbour et al., 2006). The prevalence of AML in adults is very high and it is considered the most common type of acute leukemias that occurs in adults (Lowenberg et al., 1999). The malignant transformation of the bone marrow progenitor cells in AML results in abnormal production of poorly differentiated myeloid blasts that interfere with the production of normal and functional blood cells (Lane et al., 2009; Khwaja et al., 2016). Such consequences are responsible for the characteristic symptoms of AML such as fatigue and shortness of breath due to anemia because of lowered production of RBCs, bleeding tendency due to the low number of platelets (Karpatkin, 1969; Psaila et al., 2013), and susceptibility to microbial infections due to the lack of normal functioning leukocytes (Freifeld et al., 2011). The overall survival (OS) of AML patients depends on many factors like the stage of the disease at the time of diagnosis and other health conditions. However, AML follows a rapid progression pattern and exhibit high mortality rates, where patients under the age of 60 years have an OS rate of 30–40%, while the OS is only 10% for the patients who are 60 years or older (Menzin et al., 2002; Majhail et al., 2012). 

MicroRNAs, are a class of gene regulators, whose alterations in their expression levels show close association with the onset of many diseases such as cancer. Such relationship is considered a hotspot for research, which motivated many groups to work on expression profiling of miRNAs in different cellular contexts and in different types of neoplasms (Peng and Croce, 2016; Liao et al., 2017; He et al., 2019). Interestingly, each type of leukemia had unique profile of expressed microRNA linked with specific translocation or genetic mutations (Zhang et al., 2009), and such patterns highlighted the potential to use such profiling strategy to discriminate between subtypes of leukemias as well as to predict disease progression (Chan et al., 2011; Pandita et al., 2019). Genes responsible for the production of miRNAs are first transcribed into hairpin structured precursor microRNA molecules, which then are processed to give rise into the mature miRNAs; 19-22-nt long (Bartel, 2004). The regulatory role of the mature miRNAs works through binding to complementary region in target mRNAs, which causes repression of protein translation (Francia et al., 2012).

MicroRNA profiling studies highlighted the potential of such molecules to be used as non-invasive diagnostic / prognostic markers for the detection and monitoring the progression of diseases that may portray a precise image for the underlying pathological conditions (Pogribny, 2018; Tiberio et al., 2015).

Regarding AML, several studies have showed that specific miRNAs profiles associate with different AML subtypes and different cytogenetic abnormalities (Mi et al., 2007). In addition, several microRNAs play roles in leukemogenesis process; acting as oncogenes, which accelerate the onset of malignancy, while others act as tumor suppressor molecules (Svoronos et al., 2016; Bi et al., 2018).

*MicroRNA-29 (miR-29)* family is a central epigenetic regulators that showed to have a significant role as anti-tumorigenic molecules in different forms of cancer (Iqbal et al., 2012; Li et al., 2017; Tari et al., 2018). Additionally, *miR-92a* plays a crucial role in the growth of human organs such as heart (Wong et al., 2016), lungs (Zhu et al., 2018) and immune system (Liang et al., 2016) but also the formation of blood vessels (Verma et al., 2019). The circulating levels of *mir-92a* was found to be elevated in the liver tissue of patients with hepatocellular carcinoma (Shigoka et al., 2010), while low levels of mir-92a were correlated with multiple myeloma (Yoshizawa et al., 2012).

In addition to assessing the levels of microRNAs that are associated with different types of cancers and leukemias, it is mandatory to identify target genes of these microRNA species so we can decipher the pathophysiology of leukemia and identify pathways regulated by *miR-29 / miR-92*. Several genetic-based algorithms have been used to identify target genes of miRNAs (Doran and Strauss, 2007). In case of *miR-29*, this microRNA family may target many transcription factors and anti-apoptotic genes (Si et al., 2013). Myeloid cell leukemia sequence1 (*MCL1*) has been identified as a potent target gene of *miR-29a* and *miR-92a* (Xu et al., 2014). *Mcl-1 *belongs to* Bcl-2 *family which considered an important player in regulation of apoptosis and any change in their expression levels will lead to the progression of human cancer (Thomas et al., 2010; Kadia et al., 2019). In addition, MCL1 functions in leading miR-29 targets into multiple cell types (Mott et al., 2007; Xiong et al., 2010) and *miR-29a* targets MCL1, which act as an anti-apoptotic transducer in primary AML samples (Garzon et al., 2009).

Therefore, identifying AML-specific microRNA species will help in the diagnosis of AML patients and their levels may correlate with AML pathogenesis and suggests a potential role played by these molecules in disease control. Such strategy could be achieved by quenching oncogenic miRNAs or upregulating pro-apoptotic species, via the transfection of synthetic RNA oligonucleotides representing the anti-sense or sense strands of target miRNAs, respectively (Marcucci et al., 2011).

## Materials and Methods

*Patient Criteria*

To study the modulation of microRNAs levels in AML patients, 40 newly diagnosed AML patients were enrolled in this study (14 males and 26 females, who have not received any treatment at the time of enrollment in the study; Age range 21-68), in addition to control group, which comprised of 20 healthy subjects (10 males and 10 females; Age range 19-69). The diagnosis of AML was based on the new WHO Classification based on FAB morphological subtypes and Immunophenotyping done by flow cytometry. Cytogenetic analysis was performed to detect chromosomal translocations. Mutation analysis using PCR were done to detect the presence of Internal Tandem Duplications (ITD) in *FMS- like tyrosine kinase 3 (FLT3) *gene. All patients were informed of the details of the research and signed a written consent. This study was approved by the Ethical Committee of the National Cancer Institute, Cairo University Sample Collection, Extraction of total RNA and MicroRNAs and Reverse Transcription:

Bone marrow aspiration samples and peripheral blood samples were collected from patients along with blood samples from control subjects. Plasma samples were separated from blood cells by centrifugation, transferred to separate tubes and stored at -80ºC until used for microRNAs extraction. Blood cell pellets were subjected to RBCs lysis buffer (5mm MgCl2,10mm NaCl, 10mm Tris-HCl, pH7.0; Promega, USA). The remaining leukocytes were used for total RNA extraction using Trizol reagent (ThermoFisher, USA). Total RNA samples were reverse-transcribed into cDNA using RevertAid First Strand cDNA synthesis kit (ThermoFisher, USA) following the manufacturer’s recommended instructions. The cDNA samples were used as templates to quantitatively analyze the transcript level of the target gene *(MCL1*). On the other hand, circulating mature miRNAs was isolated from plasma using miRNAeasy kit (Qiagen, Germany), which were then reverse transcribed using the miScript II RT KIT (Qiagen, Germany). 

*Quantitative polymerase chain reaction (qPCR)*

All primers used in the study were synthesized and HPLC – purified (Eurofins, Germany; Table 1).

*QPCR assay for microRNAs*

Primer sequences used for miRNAs analysis were retrieved from miRbase database. Real time PCR was used to detect *miR29a-3p* and *mir-92a-3p* levels using miScript SYBR green PCR kit (Qiagen, Germany) according to manufacturers’ recommendations. The thermal amplification profile was set as following; at 95°C for 5 min as initial denaturation step, followed by 94°C for 15 s, 55°C for 30s, and 70°C for 30s for 50 cycles. At the end of the PCR cycles, products’ melt curve data were collected. Expression levels of miRNAs were normalized to mir-16-5P. 

*QPCR assay for the target gene*

Structures of primers used for MCL1 target gene analysis (Table 2) were checked using Oligo Analyzer Software (Integrated DNA technologies, USA). In primers design, we considered to permit specific amplification and to avoid amplification off contaminating genomic DNA or formation of self-dimers, hairpin structures or heterodimers were Primers were synthesized and subjected to HPLC purification (Eurofins, Germany).Amplification of MCL1 target gene was carried out by using SYBR green dye (GoTaq qPCR master mix; Promega, USA) along with amplification of GAPDH as a house keeping gene. The total volume of the PCR Reactions was 20 µl containing 3 μl of 1:30 diluted cDNA sample (equivalent to 5 ng total RNA), 10μl 2X GoTaq^®^ qPCR Master Mix (Promega), 0.25 pmole, each of forward and reverse primers, and nuclease free water was added to bring the volume to 20 µl. All reactions were performed in duplicate and the reactions that showed inconsistency between the duplicates were repeated. The thermal profile Started with denaturation at 95°C for 5 min, followed by 50 cycles of 95°C for 15 s, 60°C for 30 s, and 72°C for 30 s. At the end of the PCR cycles, melt curves of the amplicons were obtained. In order to verify the amplification of correct products, PCR reactions were separated onto 1.5% agarose gel electrophoresis. 

*Data analysis*

The relative quantification (RQ) of the target gene expression was calculated using comparative CT method (2^-ΔΔCT^) where ΔΔCT is the difference between ΔCT values of the leukemia patient and that of the control group (ΔΔCT = Ct_target_-Ct_reference_)_AML sample_ -(Ct_target_-Ct_reference_)_Control sample._

*Statistical analysis*

Data was analyzed using SPSS software version 16 (SPSS Inc., Chicago, IL). Qualitative data were described using numbers and percentages. Quantitative data were described using median, mean and standard deviation. Comparisons between numerical variables of two groups were done by unpaired student’s t test for parametric data, Kruskal-Wallis Test was used to compare more than two groups for qualitative variables Correlation analysis: Pearson coefficient where the sign of the coefficient indicates whether the relation is positive or negative, while the value indicates the strength of the correlation as follows: weak correlation: r <0.25, intermediate correlation: 0.25-0.74 and strong correlation: 0.75-0.99 All statistical tests were two-sided, and P values less than 0.05 were considered as statistically significant.

## Results

Data presented in this study were obtained from the analysis of samples collected from 40 AML patients who were referred to the department of clinical pathology between February 2013 to October 2014. In this study, the median age of patient was 46 years. A total of 20 healthy individuals with a median age of 27 years served as the control group. In our study we used peripheral blood or bone marrow samples with significant percentage of myeloid blasts. Samples were assayed to quantify the circulating levels of *miR-29a-3p*, and *miR-92a-3p* and the transcript level of MCL1 in leukocytes by real-time PCR. 

*Hematological data*

Complete Blood count (CBC) was performed for all subjects enrolled in this study. The CBC revealed that all the AML patients suffered from severe anemia with significant reduction in Hb levels (p<0.001) (Hb ranged from 2.9-10.4 g / dl with a mean value of 7.35±1.87 g / dl. The Hb of the control group, on the other hand, ranged from 11.1-15.3 g / dl with a mean value of 12.60±1.46 g / dl. Regarding the Total Leucocytic count (TLC), data of AML patients ranged from 11.5-150.1 X10^9^/L with a mean value of 80.9±69.2 X10^9^/L, while the control group ranged from 3.3-6.4 X 10^9^/L with a mean value of 5.21±1.18 (p < 0.001). Platelet counts (PLT) also showed significant decrease in AML patients with a mean value of 37.32-38.43 X10^9^/L, while in the control group had higher levels with a mean value of 250 X10^9^/L. There is a statistically significant decrease in platelet count as compared to the control group, (p <0.001). The detailed hematologic data of AML patients are presented in Table 3.

*Morphological Analysis, Immunophenotyping, Cytogenetic Analysis and FLT3 mutations*

Bone marrow aspiration was obtained from all the patients enrolled in this study, and classification of the AML cases performed by using the FAB criteria based on morphological and cytochemical picture (M0 to M7). For morphologic inspection, blood smears were Leishman-stained analyzed. For Flow Cytometry, Bone marrow aspirates or Peripheral Blood samples were collected for surface antigen staining using different monoclonal antibodies. There was a heterogeneity in FAB subtypes in this study (data shown in Table 4). Standard cytogenetic analysis was performed to detect chromosomal abnormalities, where our data showed that 33 patients had normal karyotype while 7 patients had chromosomal translocations (Data shown in table 4). Also, in this study, Genomic DNA was screened for the presence of mutational events in the cytoplasmic region of FLT3 receptor. Internal tandem duplications in FLT3 (FLT3-ITD) was found in 9 patients while the rest 31 cases were negative for FLT3-ITD.

*Expression levels of circulating mir-29a-3p and mir-92a-3p in AML patients*

**Figure 1:**
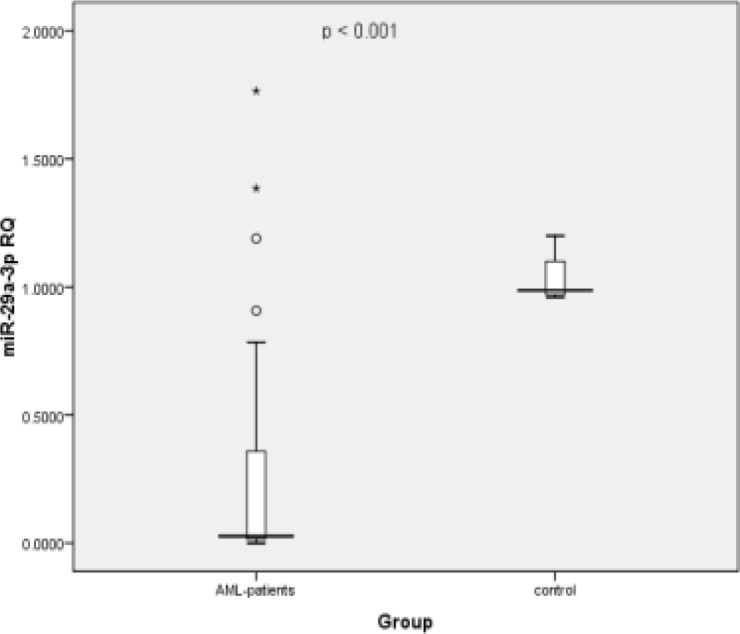
Boxplot Showing the Difference in mir-29a-3p Circulatory Plasma Levels between Control Subjects and AML Patients; Significant downregulation was observed in case of the AML patients (Minimum fold change was 2.428 and the maximum fold decrease was 2857.14).

**Figure 2:**
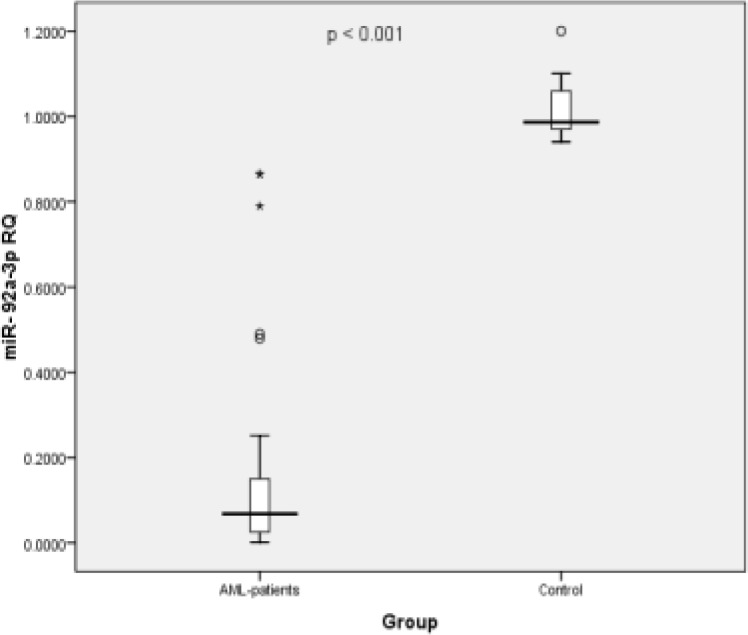
Boxplot Showing the Difference in mir-92a-3p Circulatory Plasma Levels between Control Subjects and AML Patients; Significant downregulation was observed in case of the AML patients (Minimum fold change was 2.08 and the maximum fold decrease was 609.75).

Compared to normal controls, miR-29a-3p circulating levels in the plasma of adult AML patients were significantly decreased (p < 0.001, Figure 1); this reduction was observed in 90% of the patients. Similarly, the plasma mir-92a-3p level in the patients were dramatically decreased when compared to that in healthy controls (p < 0.001, Figure 2); 100% of the patients had low levels of circulating mir-92a-3p. Using Spearman’s correlation, data analysis showed that the expression levels of circulating *mir-29a-3p* and *mir-92a-3p* in AML patient was almost the same as the patients’ bone marrow samples. Moreover, there was a statistically significant positive direct correlation between expression of *mir-29a* and *mir-92a* (R=0.533, p = 0.001) (Figure 6).

*Correlations between plasma levels of mir-29a-3p/mir-92a-3p and clinical picture*

In this study, we investigated the correlation between plasma levels of *mir-29a-3p*/*mir-92a-3p* with the clinical characteristics of adult AML patients. Regarding* miRNA 29a-3p*, there was no correlation between *miRNA 29a-3p *levels and age, TLC, PLT, cytogenetic abnormalities and FLT3 mutations. However, there was a clear significant correlation between *miRNA 29a-3p* and sex as its levels were markedly higher in female patients compared to males (p =0.014). Additionally, *mir29a-3p* expression was significantly downregulated in patients expressing CD13 (p =0.02). Regarding *miRNA 92a-3p*, there was no correlation between *miRNA 92a-3p* levels and age, sex, or hematological parameters (Hb, TLC and PLT).

*Expression levels of the target gene MCL1 in acute myeloid leukemia*

**Figure 3:**
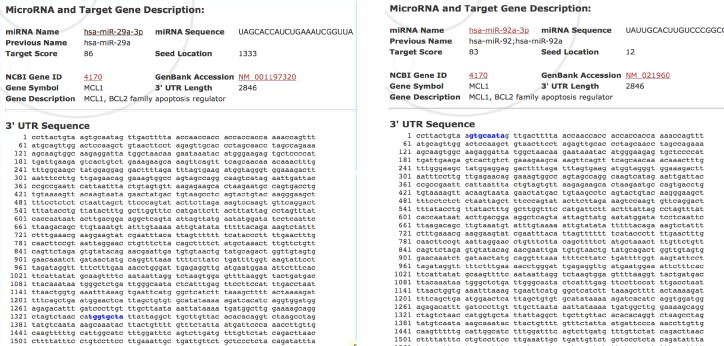
Binding Sites of MiR-29a-3p and MiR-92a-3p on MCL1 Transcript; Retrieved Using miR-DB Database

**Figure 4:**
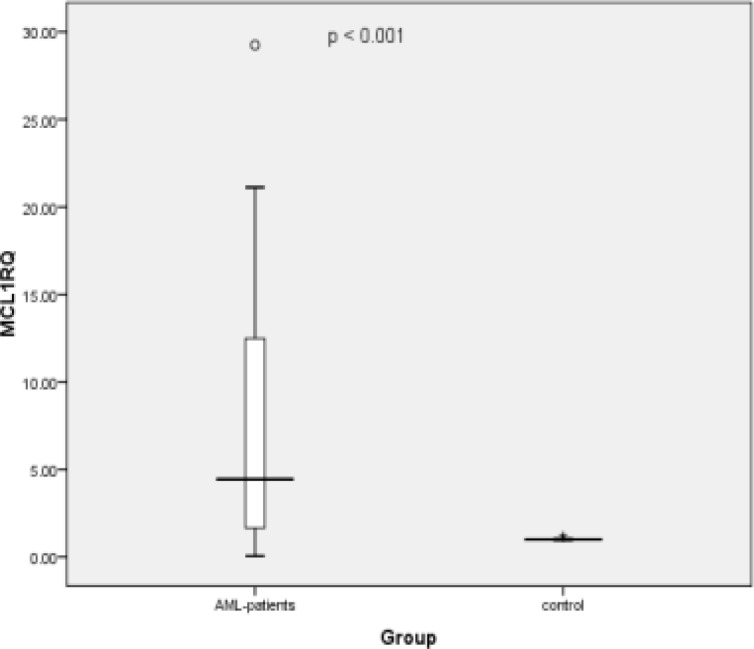
Boxplot Showing the Difference in MCL1 Levels in the Leukocytes between Control Subjects and AML Patients; Significant downregulation was observed in case of the AML patients (Minimum fold change was 2.08493 and the maximum fold increase was 29.24).

**Figure 5:**
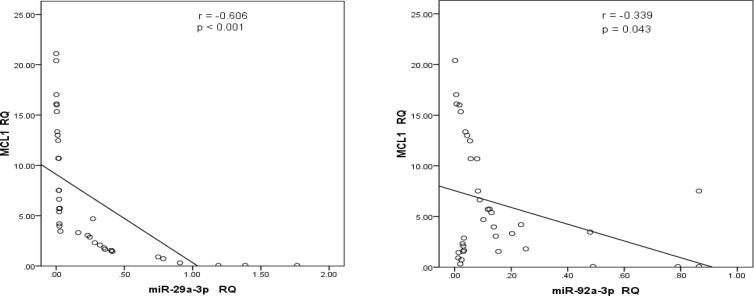
The Correlation between MCL1 Expression and mir-29a-3p and mir 92-3p Expression. Strong Negative correlation was observed in both cases (R= - 0.608 and – 0.339, p < 0.001 and 0.043 respectively).

To clarify the mechanisms underlying the roles of *miR-29a-3p* and* mir-92a-3p*, public miRNA databases. miRDB was scanned to predict the potential target of *miR-29a-3p* and *mir-92a-3p*. As indicated in Figure 3, the 3′-UTR of *MCL1* contains a binding site for both microRNAs. To confirm this relationship, we assessed the expression levels of *MCL1* in blood cells of AML patients. The data analysis revealed that *MCL1* exhibited altered expression in 95% of the patients compared to the healthy control group. The majority of the patients (77.5%) had increased levels of *MCL1 *transcripts, 17.5% of the patients showed slight downregulation in *MCL1 *levels, while the rest of the patients (5%) had comparable values to that of the control group. The elevation of RQ values of MCL1 gene transcript in the AML patients was highly significant, p < 0.001 (Figure 4). There was no correlation between *Mcl-1* expression and age, sex, TLC, PLT, BM blasts or any of the immunophenotypic markers. Regarding cytogenetic analysis, 82.5% of AML patients are within the group of the intermediate risk with Normal Karyotype (33 patients) of which 25 showed the highest statistically significant expression of* MCL1* (p <0.001), Table 4. However, the data analysis revealed that *Mcl-1 *expression was significantly decreased in patients with low Hb concentration (p =0.01). Interestingly, the correlation analysis showed a negative correlation between both *mir-29a-3p* and *mir-92a-3p* levels and *MCL1 RQ* values in AML patients (R= -0.606, p < 0.001, R= -0.339, p = 0.043, Respectively) (Figure 5). 

**Table 1:** Sequence of miRNA Primers Used in qPCR

MicroRNA	Sequence
Mir 29 -a3p	5’TAGCACCATCTGAAATCGGTTA3’
Mir 92a-3p	5’TATTGCACTTGTCCCGGCCTGT3’
Mir 16-5p	5’TAGCAGCACGTAAATATTGGCG3’

**Table 2:** Primers Used for Quantification of Target Gene

Primer	Sequence	Product size
MCL1-F	5’CGGCAGTCGCTGGAGATTATCT3’	188 bp
MCL1-R	5’TTGATGTCCAGTTTCCGAAGCAT3’	
GAPDH-F	5’CAGCCTCAAGATCATCAGCAATG3’	137 bp
GAPDH-R	5’CAGTCTTCTGGGTGGCAGTGA3’	

**Table 3:** Hematological Data of the AML Patients:

	Patients (40) Mean±SD	Controls (20) Mean±SD	P-value
Hb (gm/dl)	7.35±1.87	12.6±1.457	<0.001
WBCs (X 10^9^/L)	80.9±69.2	5.21±1.18	<0.001
Platelets (X 10^9^/L)	60.21±43.72	250.5±2.3	<0.001

P-value <0.05 is considered significant; Hb, Hemoglobin; WBC, white blood cell count

**Table 4:** Immunophenotyping and Cytogenetic Analysis of AML Patients

	Number of patients (total n=40)	Percentage
FAB subtypes		
M0	3	7.5
M1	15	37.5
M2	8	20
M3	2	5
M4	8	20
M5	2	5
RAEB-M	2	5
Immunophenotyping		
CD13+	38	95
CD33+	34	85
CD34+	18	45
CD11C+	10	25
CD4 +	6	15
CD14+	6	15
CD7+	4	10
Cytogenetics		
favorable risk		
T (8;21)	3	7.5
T (15;17)	2	5
Intermediate risk		
Normal Karyotype	33	82.5
Unfavorable risk		
T (9;22)	2	5

**Figure 6:**
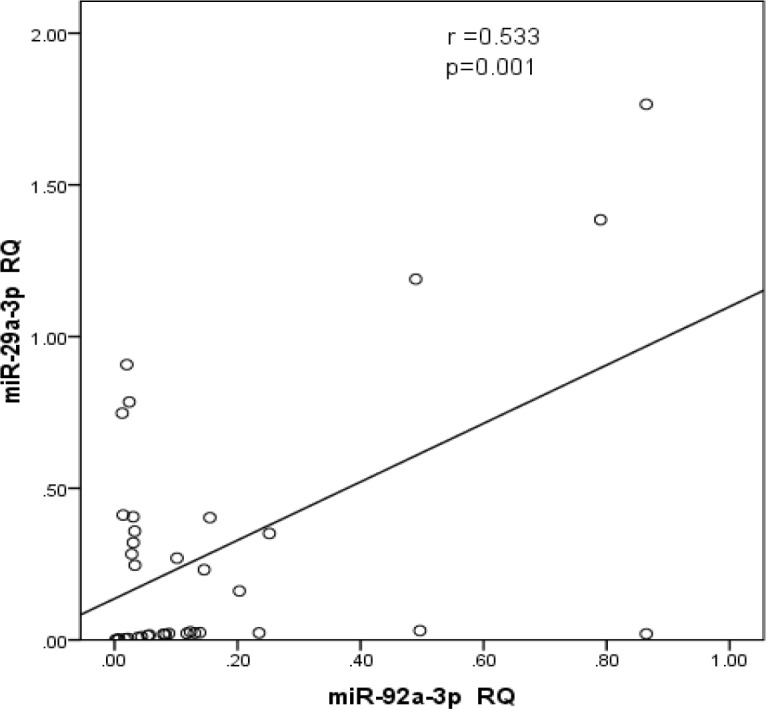
The Correlation between mir 29a-3p and mir 92a-3p Expression. Strong positive correlation was observed between both microRNA species (R=0.533 and p =0.001)

## Discussion

AML is one of the most aggressive hematopoietic malignancies that initiates at the bone marrow and leads to the aberrant production of blood cells (Dohner et al., 2010). Searching for novel markers for the early detection of AML onset is mandatory for disease management and to allow initiation of the recommended treatment, which would improve the patients’ quality of life and help in achieving complete remission or increase the overall survival of patients (Bai et al., 2013). Liquid biopsies represented by body fluids provide an important source of circulating non-invasive cancer biomarkers that can be utilized instead of the expensive and invasive traditional diagnostic methods (Palmirotta et al., 2018). Among these markers miRNAs, which act as gene regulators and their abnormal expression and dysregulation is common in many diseases and thus, they can be used for detecting pathological conditions (Soifer et al., 2007; Cui et al., 2019; Liu et al., 2019). 

The aim of our study was to assess the potential role of plasma *miR29a* and *miR-92a* levels as diagnostic and/or prognostic markers for detection of the onset and/or progression of AML in adult patients. In addition, we tried to find the clinical and prognostic implications of miR29a and *miR-92a* dysregulation. In the meantime, we aimed to validate the regulatory role of *miR29a* and *miR-92a* on the oncogenic gene, *MCL1*.

Bone marrow and Peripheral blood samples were collected from patients enrolled in this study, and microRNAs were extracted and quantified using Real-Time PCR using specific *mir29a-3p* and* mir92a-3p* primers. We observed that there is a significant downregulation of *mir29a-3p* and *mir92a-3p* in the bone marrow aspirates and the plasma of the newly diagnosed AML patients when compared to normal subjects; (p <0.001). Similar Results were reported by other study groups (Zhu et al., 2013; Gong et al., 2014; Xu et al., 2014) who reported a downregulation in *miR-29a/29b* levels in peripheral blood mononuclear cells and bone marrow from AML patients compared with healthy individuals. Also, *miR-29a* was found to be downregulated in many other types of neoplasms like lung cancer (Barkley and Santocanale, 2013), oral squamous carcinoma (Lu et al., 2014), glioblastoma (Xi et al., 2017), metastatic prostate cancer (Ahmed et al., 2013), ALK-positive anaplastic large cell lymphomas (Desjobert et al., 2011), endocrine-sensitive breast cancer (Muluhngwi et al., 2017).

This downregulation of *mir29a* is attributed to its function, which was demonstrated to play an important role in inducing apoptosis process in the cells. This downregulation is usually reached through regulation of methylation. In our study we found that *mir-29a *downregulation will lead to the overexpression of *MCL1*, an anti-apoptotic gene that helps in survival of cancer cells and inhibits apoptosis (Gong et al., 2014). *MC11* is one of the markers that always found to be associated with the pathogenesis of leukemia and also is implicated in the drug resistance process and relapse in AML (Pan et al., 2015). After Investigating the expression of *MCL1 *using qPCR, we detected that the majority of the patients (31/40, 77.5%) had upregulated *MCL1*. *Mir-29* has the ability to suppress the anti-apoptotic ability of the cells through negative regulation of the oncogenic *Matrix-Metalloproteinase 2 (MMP-2)* gene (Li and Li, 2013). Moreover, the absence of *miR-29a *will lead to the aberrant upregulation of HSP60, a member of heat shock proteins family, and downregulating other members like HSP27/40/70/90 in breast cancer as reported by Choghaei et al., (2016). In addition to the role of *miR-29a* in inducing apoptosis, it also functions as angiogeneic inhibitor which is required for malignant neoplasms. *Mir-29a* regulates and suppresses the expression of vasohibin 2 (VASH2); one of the angiogenic factors (Jia et al., 2016). Interestingly, previous studies reported that treatment of leukemia with *miR-29a* prevents the proliferation of myeloid blasts (Garzon et al., 2009; Han et al., 2010; Amodio et al., 2015). 

On the contrary, some studies showed that AML patients had an elevated level of *miR-29a* (Wang et al., 2012). Many reports showed that over expression of *miR-29a* will lead to increased cellular proliferation through suppressing the expression of Mucin1, as well as regulating MAPK and β-catenin pathways (Trehoux et al., 2015). Another study by Han et al., (2010) showed that overexpression of miR-29a in blood cells resulted in the onset of myeloproliferative neoplasms that can be developed to give AML. In addition, *miR-29a* mediates progenitor cells proliferation by accelerating the transition of G1 phase to S/G2 phase (Amodio et al., 2015). Furthermore, different members of miR-29 family were overexpressed in sera of patients with osteosarcoma, colorectal cancer (Huang et al., 2010; Brunet Vega et al., 2013) and in metastatic-liver carcinoma (Wang and Gu, 2012) as well as in breast cancer (Wu et al., 2012).

In addition to mir-29a quantification, we also estimated the levels of plasma *mir-92a-3p* in AML patients, and the data analysis revealed that there is an obvious downregulation in the level of this microRNA species compared to healthy subjects (p < 0.001). As reported previously, in AML cell lines, *miR-92a* was remarkably downregulated and consequently leads to the over-expression of *Methylenetetrahydrofolate dehydrogenase 2 (MTHFD2)* leading to increasing in the propagation and survival of malignant cells (Gu et al., 2017) .

Our results are in accordance with many other studies (Tanaka et al., 2009; Elhamamsy et al., 2017) who reported that* mir-92a* plasma levels were significantly lower in AML patients and also it was found to be very low in tumor tissues retrieved from patients with gastric cancer (Zhao et al., 2018), However, other studies found that suppression of *miR-92a* will induce apoptosis and can be considered as a novel method for treatment of Acute Promyelocytic Leukemia (Sharifi et al., 2014). Also, upregulation in miR-92a was observed in colorectal cancer (Elshafei et al., 2017) and cervical cancer (Zhou et al., 2015; Kawai et al., 2018).

In our study we could not find significant correlation between the levels of *mir29a-3p* and *mir92a-3p* expression and AML-subtypes, however, Zhu et al., (2013) studied the association of miR-29a levels in pediatric acute leukemia and they found that *miR-29a *was downregulated in AML-M7 compared to other subtypes (p <0.001). However, one of our future perspectives is to increase the sample size and recruit more patients with various AML-subtypes which may help in establishing a real subtype-dependent relationship. The authors evaluated the levels of *miR-29a* in patients with different cytogenetic analysis and they found that high *miR-29a* levels were detected among patients with favorable karyotypes (Zhu et al., 2013). Similarly, El-Halawani et al., (2017) found that the *mir92a *levels were significantly lower in the poor risk cytogenetics as compared to the favorable and intermediate risk cytogenetics. In addition to the lack of correlation of the tested miRNAs with subtypes and karyotypes, we also could not find significant relationship with FLT3-ITD. However, El-Halawani et al., (2017) reported high level of *miRNA29a* expression in Flt3-ITD+ as compared to the Flt3-ITD. Moreover, the correlation analysis revealed the presence of significant positive direct association between levels of *mir-29a* and *mir-92a* (r=0.533, p = 0.001) and negative correlation with MCL1 transcript level was observed (r= -0.606, p<0.001, r= -0.339, p=0.043, respectively) and this was consistent with other studies that showed that down-regulation of* miR-29a* and *miR-29b* accompanied by up-regulation of the anti-apoptotic gene *MCL1* transcripts as well as *Bcl*_2_ in myeloid leukemias and such increase reflects poor prognosis (Li et al., 2019). In our study, no relationship was found between* Mcl-1* expression and age, sex, TLC, PLT, BM blasts, cell surface antigens or FLT3-ITD. However, *Mcl-1* expression showed a considerable decrease in patients with low Hb concentration and in patients with Normal karyotype (p<0.05 and 0.01, respectively). 

In conclusion, we investigated the circulating levels of *mir-29a-3p/mir-92a-3p* in AML patients and we found that both species were downregulated in the patients’ plasma and they are negatively correlated with their target gene MCL1 transcript levels. The data proved our hypothesis that *mir-29a* and* mir-92a *act as anti-tumorigenic molecules and highlighted their potential as promising non-invasive diagnostic biomarker for AML that can be utilized in the future as candidates for therapeutic intervention.

## References

[B1] Ahmed F, Shiraishi T, Vessella RL (2013). Tumor necrosis factor receptor associated factor-4: an adapter protein overexpressed in metastatic prostate cancer is regulated by microRNA-29a. Oncol Rep.

[B2] Amodio N, Rossi M, Raimondi L (2015). miR-29s: a family of epi-miRNAs with therapeutic implications in hematologic malignancies. Oncotarget.

[B3] Bai J, He A, Zhang W (2013). Potential biomarkers for adult acute myeloid leukemia minimal residual disease assessment searched by serum peptidome profiling. Proteome Sci.

[B4] Barkley LR, Santocanale C (2013). MicroRNA-29a regulates the benzo[a]pyrene dihydrodiol epoxide-induced DNA damage response through Cdc7 kinase in lung cancer cells. Oncogenesis.

[B5] Bartel DP (2004). MicroRNAs: genomics, biogenesis, mechanism, and function. Cell.

[B6] Bi L, Sun L, Jin Z (2018). MicroRNA-10a/b are regulators of myeloid differentiation and acute myeloid leukemia. Oncol Lett.

[B7] Brunet Vega A, Pericay C, Moya I (2013). microRNA expression profile in stage III colorectal cancer: circulating miR-18a and miR-29a as promising biomarkers. Oncol Rep.

[B8] Chan E, Prado DE, Weidhaas JB (2011). Cancer microRNAs: from subtype profiling to predictors of response to therapy. Trends Mol Med.

[B9] Choghaei E, Khamisipour G, Falahati M (2016). Knockdown of microRNA-29a changes the expression of heat shock proteins in breast carcinoma MCF-7 cells. Oncol Res.

[B10] Cui M, Wang H, Yao X (2019). Circulating MicroRNAs in Cancer: Potential and challenge. Front Genet.

[B11] Desjobert C, Renalier MH, Bergalet J (2011). MiR-29a down-regulation in ALK-positive anaplastic large cell lymphomas contributes to apoptosis blockade through MCL-1 overexpression. Blood.

[B12] Dohner H, Estey EH, Amadori S (2010). Diagnosis and management of acute myeloid leukemia in adults: recommendations from an international expert panel, on behalf of the European LeukemiaNet. Blood.

[B13] Doran J, Strauss WM (2007). Bio-informatic trends for the determination of miRNA-target interactions in mammals. DNA Cell Biol.

[B14] Elhamamsy AR, El Sharkawy MS, Zanaty AF (2017). Circulating miR-92a, miR-143 and miR-342 in plasma are novel potential biomarkers for acute myeloid leukemia. Int J Mol Cell Med.

[B15] Elshafei A, Shaker O, Abd El-Motaal O (2017). The expression profiling of serum miR-92a, miR-375, and miR-760 in colorectal cancer: An Egyptian study. Tumour Biol.

[B16] Francia S, Michelini F, Saxena A (2012). Site-specific DICER and DROSHA RNA products control the DNA-damage response. Nature.

[B17] Freifeld AG, Bow EJ, Sepkowitz KA (2011). Clinical practice guideline for the use of antimicrobial agents in neutropenic patients with cancer: 2010 Update by the Infectious Diseases Society of America. Clin Infect Dis.

[B18] Garzon R, Heaphy CE, Havelange V (2009). MicroRNA 29b functions in acute myeloid leukemia. Blood.

[B19] Gong JN, Yu J, Lin HS (2014). The role, mechanism and potentially therapeutic application of microRNA-29 family in acute myeloid leukemia. Cell Death Differ.

[B20] Gu Y, Si J, Xiao X (2017). miR-92a inhibits proliferation and induces apoptosis by regulating methylenetetrahydrofolate dehydrogenase 2 (MTHFD2) expression in acute myeloid leukemia. Oncol Res.

[B21] Han YC, Park CY, Bhagat G (2010). microRNA-29a induces aberrant self-renewal capacity in hematopoietic progenitors, biased myeloid development, and acute myeloid leukemia. J Exp Med.

[B22] He C, Luo B, Jiang N (2019). OncomiR or antioncomiR: Role of miRNAs in acute myeloid leukemia. Leuk Lymphoma.

[B23] Huang Z, Huang D, Ni S (2010). Plasma microRNAs are promising novel biomarkers for early detection of colorectal cancer. Int J Cancer.

[B24] Iqbal J, Shen Y, Liu Y (2012). Genome-wide miRNA profiling of mantle cell lymphoma reveals a distinct subgroup with poor prognosis. Blood.

[B25] Jabbour EJ, Estey E, Kantarjian HM (2006). Adult acute myeloid leukemia. Mayo Clin Proc.

[B26] Jia P, Cai H, Liu X (2016). Long non-coding RNA H19 regulates glioma angiogenesis and the biological behavior of glioma-associated endothelial cells by inhibiting microRNA-29a. Cancer Lett.

[B27] Kadia TM, Kantarjian HM, Konopleva M (2019). Myeloid cell leukemia-1 dependence in acute myeloid leukemia: a novel approach to patient therapy. Oncotarget.

[B28] Karpatkin S (1969). Heterogeneity of human platelets Metabolic and kinetic evidence suggestive of young and old platelets. J Clin Invest.

[B29] Kawai S, Fujii T, Kukimoto I (2018). Identification of miRNAs in cervical mucus as a novel diagnostic marker for cervical neoplasia. Sci Rep.

[B30] Khwaja A, Bjorkholm M, Gale RE (2016). Acute myeloid leukaemia. Nat Rev Dis Primers.

[B31] Lane SW, Scadden DT, Gilliland DG (2009). The leukemic stem cell niche: current concepts and therapeutic opportunities. Blood.

[B32] Li L, Li H (2013). Role of microRNA-mediated MMP regulation in the treatment and diagnosis of malignant tumors. Cancer Biol Ther.

[B33] Li XX, Zhou JD, Wen XM (2019). Increased MCL-1 expression predicts poor prognosis and disease recurrence in acute myeloid leukemia. Onco Targets Ther.

[B34] Li Y, Wang Z, Li Y (2017). MicroRNA-29a functions as a potential tumor suppressor through directly targeting CDC42 in non-small cell lung cancer. Oncol Lett.

[B35] Liang DY, Hou YQ, Luo LJ (2016). Altered expression of miR-92a correlates with Th17 cell frequency in patients with primary biliary cirrhosis. Int J Mol Med.

[B36] Liao Q, Wang B, Li X (2017). miRNAs in acute myeloid leukemia. Oncotarget.

[B37] Liu Y, Cheng Z, Pang Y (2019). Role of microRNAs, circRNAs and long noncoding RNAs in acute myeloid leukemia. J Hematol Oncol.

[B38] Lowenberg B, Downing JR, Burnett A (1999). Acute myeloid leukemia. N Engl J Med.

[B39] Lu L, Xue X, Lan J (2014). MicroRNA-29a upregulates MMP2 in oral squamous cell carcinoma to promote cancer invasion and anti-apoptosis. Biomed Pharmacother.

[B40] Majhail NS, Brazauskas R, Hassebroek A (2012). Outcomes of allogeneic hematopoietic cell transplantation for adolescent and young adults compared with children and older adults with acute myeloid leukemia. Biol Blood Marrow Transplant.

[B41] Marcucci G, Mrozek K, Radmacher MD (2011). The prognostic and functional role of microRNAs in acute myeloid leukemia. Blood.

[B42] Menzin J, Lang K, Earle CC (2002). The outcomes and costs of acute myeloid leukemia among the elderly. Arch Intern Med.

[B43] Mi S, Lu J, Sun M (2007). MicroRNA expression signatures accurately discriminate acute lymphoblastic leukemia from acute myeloid leukemia. Proc Natl Acad Sci U S A.

[B44] Mott JL, Kobayashi S, Bronk SF (2007). mir-29 regulates Mcl-1 protein expression and apoptosis. Oncogene.

[B45] Muluhngwi P, Krishna A, Vittitow SL (2017). Tamoxifen differentially regulates miR-29b-1 and miR-29a expression depending on endocrine-sensitivity in breast cancer cells. Cancer Lett.

[B46] Palmirotta R, Lovero D, Cafforio P (2018). Liquid biopsy of cancer: a multimodal diagnostic tool in clinical oncology. Ther Adv Med Oncol.

[B47] Pan R, Ruvolo VR, Wei J (2015). Inhibition of Mcl-1 with the pan-Bcl-2 family inhibitor (-)BI97D6 overcomes ABT-737 resistance in acute myeloid leukemia. Blood.

[B48] Pandita A, Ramadas P, Poudel A (2019). Differential expression of miRNAs in acute myeloid leukemia quantified by Nextgen sequencing of whole blood samples. PLoS One.

[B49] Peng Y, Croce CM (2016). The role of MicroRNAs in human cancer. Signal Transduct Target Ther.

[B50] Pogribny IP (2018). MicroRNAs as biomarkers for clinical studies. Exp Biol Med (Maywood).

[B51] Psaila B, Bussel JB, Frelinger AL (2013). Differences in platelet function in patients with acute myeloid leukemia and myelodysplasia compared to equally thrombocytopenic patients with immune thrombocytopenia: a reply to a rebuttal. J Thromb Haemost.

[B52] Sharifi M, Salehi R, Gheisari Y (2014). Inhibition of microRNA miR-92a induces apoptosis and necrosis in human acute promyelocytic leukemia. Adv Biomed Res.

[B53] Shigoka M, Tsuchida A, Matsudo T (2010). Deregulation of miR-92a expression is implicated in hepatocellular carcinoma development. Pathol Int.

[B54] Si H, Sun X, Chen Y (2013). Circulating microRNA-92a and microRNA-21 as novel minimally invasive biomarkers for primary breast cancer. J Cancer Res Clin Oncol.

[B55] Soifer HS, Rossi JJ, Saetrom P (2007). MicroRNAs in disease and potential therapeutic applications. Mol Ther.

[B56] Svoronos AA, Engelman DM, Slack FJ (2016). OncomiR or Tumor Suppressor? The Duplicity of MicroRNAs in Cancer. Cancer Res.

[B57] Tanaka M, Oikawa K, Takanashi M (2009). Down-regulation of miR-92 in human plasma is a novel marker for acute leukemia patients. PLoS One.

[B58] Tari K, Shamsi Z, Reza Ghafari H (2018). The role of the genetic abnormalities, epigenetic and microRNA in the prognosis of chronic lymphocytic leukemia. Exp Oncol.

[B59] Thomas LW, Lam C, Edwards SW (2010). Mcl-1; the molecular regulation of protein function. FEBS Lett.

[B60] Tiberio P, Callari M, Angeloni V (2015). Challenges in using circulating miRNAs as cancer biomarkers. Biomed Res Int.

[B61] Trehoux S, Duchene B, Jonckheere N (2015). The MUC1 oncomucin regulates pancreatic cancer cell biological properties and chemoresistance Implication of p42-44 MAPK, Akt, Bcl-2 and MMP13 pathways. Biochem Biophys Res Commun.

[B62] Verma M, Asakura Y, Asakura A (2019). Inhibition of microRNA-92a increases blood vessels and satellite cells in skeletal muscle but does not improve duchenne muscular dystrophy-related phenotype in mdx mice. Muscle Nerve.

[B63] Wang LG, Gu J (2012). Serum microRNA-29a is a promising novel marker for early detection of colorectal liver metastasis. Cancer Epidemiol.

[B64] Wang XS, Gong JN, Yu J (2012). MicroRNA-29a and microRNA-142-3p are regulators of myeloid differentiation and acute myeloid leukemia. Blood.

[B65] Wong LL, Wang J, Liew OW (2016). MicroRNA and heart failure. Int J Mol Sci.

[B66] Wu Q, Wang C, Lu Z (2012). Analysis of serum genome-wide microRNAs for breast cancer detection. Clin Chim Acta.

[B67] Xi Z, Wang P, Xue Y (2017). Overexpression of miR-29a reduces the oncogenic properties of glioblastoma stem cells by downregulating Quaking gene isoform 6. Oncotarget.

[B68] Xiong Y, Fang JH, Yun JP (2010). Effects of microRNA-29 on apoptosis, tumorigenicity, and prognosis of hepatocellular carcinoma. Hepatology.

[B69] Xu L, Xu Y, Jing Z (2014). Altered expression pattern of miR-29a, miR-29b and the target genes in myeloid leukemia. Exp Hematol Oncol.

[B70] Yoshizawa S, Ohyashiki JH, Ohyashiki M (2012). Downregulated plasma miR-92a levels have clinical impact on multiple myeloma and related disorders. Blood Cancer J.

[B71] Zhang H, Luo XQ, Zhang P (2009). MicroRNA patterns associated with clinical prognostic parameters and CNS relapse prediction in pediatric acute leukemia. PLoS One.

[B72] Zhao X, Hou Y, Tuo Z (2018). Application values of miR-194 and miR-29 in the diagnosis and prognosis of gastric cancer. Exp Ther Med.

[B73] Zhou C, Shen L, Mao L (2015). miR-92a is upregulated in cervical cancer and promotes cell proliferation and invasion by targeting FBXW7. Biochem Biophys Res Commun.

[B74] Zhu C, Wang Y, Kuai W (2013). Prognostic value of miR-29a expression in pediatric acute myeloid leukemia. Clin Biochem.

[B75] Zhu Q, Zang Q, Jiang ZM (2018). Enhanced expression of non coding miR 92a expression is implicated in the development of lung cancer. Eur Rev Med Pharmacol Sci.

